# School Climate and School Identification as Determinants of Internet Gaming Disorder Among Chinese Adolescent Internet Gamers: Cross-Sectional Mediation Study

**DOI:** 10.2196/50418

**Published:** 2024-11-12

**Authors:** Yanqiu Yu, Stefanie H Y Yen, Deborah Baofeng Wang, Anise M S Wu, Juliet Honglei Chen, Guohua Zhang, Mengni Du, Dajin Du, Mingxuan Du, Joseph T F Lau

**Affiliations:** 1 School of Public Health, Fudan University, Shanghai, China; 2 Center for Health Behaviours Research, Jockey Club School of Public Health and Primary Care, The Chinese University of Hong Kong, Hong Kong, China (Hong Kong); 3 Zhejiang Provincial Clinical Research Center for Mental Disorders, The Affiliated Wenzhou Kangning Hospital, Wenzhou Medical University, Wenzhou, China; 4 Department of Psychology, Faculty of Social Sciences, University of Macau, Macao, China; 5 Centre for Cognitive and Brain Sciences, Institute of Collaborative Innovation, University of Macau, Macao, China; 6 Department of Psychology, Zhejiang Sci-Tech University, Hangzhou, China; 7 School of Mental Health, Wenzhou Medical University, Ouhai District, Wenzhou, 325000, China, +86 577 8668 9810; 8 Teaching and Research Center, Bureau of Education, Linhai, China

**Keywords:** school climate, school identification, adolescent, structural equation modeling, internet gaming disorder

## Abstract

**Background:**

School climate and school identification are important features of the school environment and potential determinants of adolescent internet gaming disorder (IGD).

**Objective:**

This novel study investigated their joint effects on IGD and related mediation mechanisms via the interpersonal factor of teacher-student relationship and the individual factors of academic stress and anxiety.

**Methods:**

A large-scale cross-sectional study was conducted among adolescent internet gamers of junior, senior, and vocational middle schools in Taizhou City, China, from February to March 2022 (N=5778). Participants self-administered an anonymous, structured questionnaire in classrooms. Adjusted logistic regression and structural equation modeling (SEM) were used for data analysis.

**Results:**

Among all participants, the prevalence of IGD was 8% (461/5778). The 4 school climate subscales (student-student relationship subscale: adjusted odds ratio [ORa] 0.88, 95% CI 0.85-0.91; student-staff relations subscale: ORa 0.87, 95% CI 0.84-0.90; academic emphasis subscale: ORa 0.88, 95% CI 0.85-0.91; shared values approach: ORa 0.88, 95% CI 0.85-0.90), the school identification subscale (ORa 0.85, 95% CI 0.83-0.88), and teacher-student relationship (ORa 0.80, 95% CI 0.76-0.84) were significant protective factors against IGD, while academic stress (ORa 1.18, 95% CI 1.14-1.23) and anxiety (ORa 1.16, 95% CI 1.14-1.18) were risk factors of IGD. The SEM showed that the negative associations between school climate and IGD and between school identification and IGD were mediated via (1) three 2-step paths, each involving a single mediator—teacher-student relationship, academic stress, and anxiety, respectively—and (2) two 3-step paths involving 2 mediators—teacher-student relationship and academic stress first, respectively, and then anxiety. The direct effect of school climate on IGD was statistically nonsignificant (ie, full mediation with effect size ranging from 4.2% to 20.4%), while that of school identification was statistically significant (ie, partial mediation with effect size ranging from 4.5% to 38.2%).

**Conclusions:**

The relatively high prevalence of IGD among Chinese adolescents may be reduced through school-based interventions to improve school climate and school identification. Such improvements may reduce the levels of risk factors of IGD (poor teacher-student relationship, academic stress, and anxiety) and hence the risk of IGD. Future longitudinal and intervention studies are needed to confirm the findings.

## Introduction

Internet gaming disorder (IGD) was included in the *International Classification of Diseases, 11th Revision (ICD-11*) as a subtype of gaming disorder by the World Health Organization [1]. Extant literature has documented the detrimental effects of adolescent IGD, including various psychological problems (eg, depression) [2-4], physical problems (eg, sleep problems) [2,5,6], interpersonal problems (eg, social anxiety) [4,7], and behavioral problems (eg, physical violence) [8]. The prevalence of adolescent IGD ranged from 0.7% to 27.5% globally [9,10] and from 13.0% to 23.8% in China [6,11,12]. Notably, these studies used different measurement tools.

The Social Cognitive Theory (SCT) postulates that environmental factors, personal characteristics, and health-related behaviors would interact with each other, a concept known as reciprocal determinism [13]. School is an important social environment and a setting for the effective promotion of adolescent health [14]. School climate reflects a salient aspect of the social environment shaping the schools’ psychosocial atmospheres and interpersonal relationships [15]. Favorable school climate was negatively associated with IGD [16-18] and problematic internet use [19,20] among Chinese adolescents. School identification refers to the important sense of belonging to the school and caring about the school’s goals [21]; it was developed via an internalization process from extrinsic motivations (eg, acknowledgment from teachers and peers) to intrinsic drives fostering belongingness [21]. School identification was associated with academic achievement [15], delinquent behaviors (eg, bullying) [22], and IGD [23] among adolescents. While school climate reflects a “group concept,” school identification represents a “me concept” [24]. Investigating these 2 indicators simultaneously could understand better how the environmental (school climate) and individual-level (school identification) factors embedded in the school environment affect adolescents’ addictive behaviors (eg, IGD).

The present study used the socioecological model [25] as the conceptual framework. It postulates that factors at environmental, interpersonal, and individual levels are all important determinants of health-related behaviors and outcomes [25]. In school settings, school climate, teacher-student relationships, school identification, and perceived academic stress and anxiety are salient examples of factors at such 3 levels, respectively. Perceived academic stress is an important risk factor of mental health (eg, depression) [26] and addictive behaviors including IGD [27]. This construct has special relevance to the Chinese culture and some Asian cultures, as academic achievement is often seen by parents as the most important ladder for upward social mobility [28]. Parental pressure for academic success among adolescents has been phenomenal and has become a common and strong stressor among adolescents [29]. Regarding the importance of the teacher-student relationship, adolescents need their significant others’ positive appraisals and recognition when developing their self-image and self-affirmation [30]. To adolescents, teachers are certainly one of the most influential sources of stress, support, and recognition [30]. A poor teacher-student relationship was common and associated with IGD [18,27]. Previous studies found a high prevalence of general anxiety disorder among adolescents in China (16%-35%) [31]. Anxiety is a predictor of IGD [32,33]. Perceived academic stress, poor teacher-student relationships, and anxiety are, hence, risk factors of IGD.

More research is needed to understand how the school environment may affect IGD. Specifically, it is important to examine the potential mechanisms in order to understand the relationships between school climate or school identification and IGD. To our knowledge, only 3 studies have looked at such mechanisms; the significant mediators included deviant peer affiliation [18], weakened cognitive function [27], and psychological insecurity [20]. No studies have looked at mediations between school identification and IGD. In this study, it was contended that perceived academic stress, teacher-student relationships, and anxiety were potential mediators, with empirical support. Favorable school climate was negatively associated with perceived academic stress [34-36]. School attachment, which is closely related to school identification [37], was positively associated with the teacher-student relationship [38]. Poor school climate and poor school identification may elevate anxiety among adolescents [39,40]. As aforementioned, these 3 potential mediators were associated with IGD.

It is plausible that anxiety would further mediate between perceived academic stress and IGD, as a review demonstrated a consistently positive association between academic stress and anxiety among Chinese students [41], and as mentioned, anxiety predicted IGD. As indirect support to this claim, a previous study found that psychological insecurity, which may cause anxiety, mediated between school climate and problematic internet use [20]. Similarly, a good teacher-student relationship may reduce anxiety [19,42]. Thus, some serial mediations may exist between school climate or school identification and IGD.

This study investigated the prevalence of IGD and its associated factors, including (1) school climate (an environmental factor) and school identification (an individual-level factor), (2) perceived academic stress and anxiety (individual-level factors), and (3) the teacher-student relationship (interpersonal factors) among middle school students who were internet gamers in a Chinese city. First, it was hypothesized that a good school climate, school identification, and teacher-student relationship would be negatively associated with IGD, while academic stress and anxiety would be positively associated with IGD. Second, some mediation hypotheses were tested: (1) three paths, each involving a single mediator (ie, perceived academic stress, teacher-student relationship, and anxiety, respectively) between school climate and IGD; (2) two serial mediation paths between school climate and IGD (good school climate → less academic stress → lower anxiety → lower IGD and good school climate → better teacher-student relationship → lower anxiety → lower IGD); (3) 5 identical paths (the three single-mediator mediations and the 2 serial mediations) between school identification and IGD, with school climate being replaced by school identification.

## Methods

### Study Design, Participants, and Setting

This cross-sectional survey was conducted among students of 5 junior middle schools, 3 senior middle schools, and 1 vocational school from February to March 2022 in Taizhou City, Zhejiang Province, China. The city has a population size of 6.1 million. Only those having played internet games in the past 12 months were included in this study. The 9 participating schools were selected conveniently. All grade 1 and grade 2 students of these schools were invited to participate in this study. With the assistance of well-trained field-workers, students self-administered a structured questionnaire in classrooms; the survey was anonymous in nature, and schoolteachers were absent to reduce external influences for students. The field-workers briefed the students about the content and objectives of this study and mentioned that completion of the questionnaire would imply informed consent. It was guaranteed that the students had the right to quit the survey at any time without facing any negative consequences. Such information was also included in the introductory statements of the questionnaire. Parents were informed about this survey, and parental opt-out was exercised, although no opt-out form was returned to the teachers. No incentives were given to the participants.

A total of 8285 students returned their completed questionnaires to the research team; 114 (1.4%) were excluded due to low quality (ie, over 20% of the questions involved missing responses), 615 (7.4%) were excluded due to logical inconsistencies between some item responses, and 1778 (23.5%) were removed from data analysis as the participants had not played internet games in the past 12 months. The final sample size for data analysis was 5778 (76.5%).

### Ethical Considerations

In this study, parents were informed about the survey, and parental opt-out was exercised. In addition, students were clearly prebriefed by the field-workers about the anonymous and voluntary nature of this study and that the submission of a completed questionnaire would indicate informed consent; such information was also printed on the cover page of the questionnaire. No written informed consent was obtained to maintain anonymity. No incentive was provided to the students. This project and the above informed consent procedures were approved by the Survey and Behavioural Research Ethics Committee of the corresponding author’s affiliated institution in 2021 (No. KNLL-20211011002).

### Studied Variables and Measurements

#### Background Information

Such information included age, school type (junior middle school, senior middle school, and vocational high school), gender, hometown outside Taizhou, whether living with both parents, and father’s and mother’s educational levels.

#### IGD Classification

The 9-item *Diagnostic and Statistical Manual of Mental Disorders, Fifth Edition* (*DSM-5*) checklist [43] assessed 9 cognitive (eg, preoccupation), affective (eg, withdrawal), and behavioral (eg, deception) symptoms of IGD that occurred in the past 12 months. The items were rated with binary yes/no response options. The endorsement of at least 5 symptoms was classified as an IGD case, which was used as a binary dependent variable in this study. The Chinese version has been validated among adolescents with satisfactory psychometric properties [44]. In this study, the Cronbach α of the checklist was 0.70.

#### School Climate and School Identification

The 15-item Abbreviated Version of the Dual School Climate and School Identification Measure—Student (SCASIM-St15) was used for assessment [45]. Validation of its Chinese version among Chinese adolescents found satisfactory psychometric properties [23]. School climate has 4 first-order factors (student-student relations, staff-student relations, academic emphasis, and shared values and approach), while school identification has another single factor. Sample items were “Student treat each other with respect” for the factor of student-student relations, “Staff goes out of their way to help students” for the factor of staff-student relations, “Teachers challenge students to do better” for the factor of academic emphasis, “There is a sense that we are all on the same team” for the factor of shared values and approach, and “I am happy to be a part of this school” for the factors of school identification. All the subscales were rated by using 5-point Likert scales, ranging from 1=strongly disagree to 5=strongly agree; higher scores indicated higher levels of corresponding constructs of school climate and school identification (continuous variables). In this study, the Cronbach α of the 5 subscales ranged from 0.89 to 0.96.

#### Teacher-Student Relationship

The scale item was “In general, how are your relationships with schoolteachers?” It was rated by using 11-point Likert scales, ranging from 0=extremely poor to 10=extremely good; a higher score indicated a better teacher-student relationship (a continuous variable).

#### Academic Stress

The scale item was “How much stress do you perceive in the academic domain?” It was rated by using 11-point Likert scales, ranging from 0=none at all to 10=extremely high; a higher score indicated a higher level of academic stress (a continuous variable).

#### Anxiety

The 7-item Generalized Anxiety Disorder Questionnaire assessed the frequency of occurrence of anxiety symptoms in the past 2 weeks [46]. Its Chinese version has been validated among adolescents in China; the psychometric properties were satisfactory [47]. A sample item was “Not being able to stop or control worrying.” The items were rated by using 4-point Likert scales, ranging from 0=not at all to 3=nearly every day; higher scores indicated higher levels of anxiety (a continuous variable). In this study, the Cronbach α of the scale was 0.93.

### Sample Size Planning

The sample size planning was conducted by using the module of Tests of Mediation Effect in Logistic Regression in PASS 2021 (NCSS LLC). Assuming a power of 0.80, an α of 0.05, a regression coefficient of 0.20 (small effect size) for the mediators, correlations of 0.20 (small effect size) for both the independent variable and the mediator, an SD of 1 for the mediator, and a marginal prevalence of IGD of 0.10, the required sample size was estimated to be 2274. The sample size of this study (n=5578) was hence deemed to be adequate.

### Statistical Analysis

Pearson correlation coefficients were generated to assess the correlations among the studied variables. Univariate logistic regression analyses were conducted to test the individual associations between each background factor or independent variable and IGD. Multivariable logistic regression analyses which adjusted for the background factors were then conducted to test the significance of the associations between the potential factors and IGD. The structural equation modeling (SEM) method using the weighted least square mean and variance adjusted estimation was performed to test potential single-mediator mediations and serial mediations involving school climate or school identification, the teacher-student relationship, academic stress, anxiety, and IGD, after adjusting for the background factors. A latent variable of school climate was created from the subscale scores of the 4 factors of school climate according to the SCASIM-St15. Recommended goodness-of-fit statistics and cutoff criteria of SEM were both a comparative fit index (CFI) and Tucker-Lewis index (TLI) of ≥0.90 [48]. The SEM was conducted by using Mplus 7.0 (Muthén & Muthén), while the other tests were analyzed by SPSS 23.0 (IBM Corp). Statistical significance was defined as *P*<.05 (2-tailed tests).

## Results

### Descriptive Statistics

Of all 5778 participants, the mean (SD; range) age was 14.9 (1.5; 10‐20) years; the proportions of students of junior middle schools, senior middle schools, and vocational high schools were 59.6% (n=3445), 32.7% (n=1890), and 7.7% (n=443), respectively. Over half were male (n=3606, 62.4%), 16.9% (n=977) had their hometowns outside Taizhou, and over one-quarter did not live with both parents (n=1607, 27.8%). About one-tenth of the participants’ fathers (n=526, 9.1%) and mothers (n=529, 9.2%) had attained education in college or above. The prevalence of IGD was 8% (n=461; see Table 1). The mean (SD; range) scores of the scales and subscales regarding school climate, school identification, the teacher-student relationship, academic stress, and anxiety are presented in Table 2.

**Table 1.** Participant characteristics.

	n	%
Overall	5778	100
**Background factors**		
**School type**		
Junior middle school	3445	59.6
Senior middle school	1890	32.7
Vocational high school	443	7.7
**Gender**		
Female	2133	36.9
Male	3606	62.4
Missing data	39	0.7
**Living at the studied city constantly**		
Yes	4752	82.2
No	977	16.9
Missing data	49	0.8
**Living with both parents**		
Yes	4145	71.7
No	1607	27.8
Missing data	26	0.4
**Father’s educational level**		
Junior middle school or below	3766	65.2
Senior middle school/vocational high school	1345	23.3
College or above	526	9.1
Missing data	141	2.4
**Mother’s educational level**		
Junior middle school or below	4006	69.3
Senior middle school/vocational high school	1066	18.4
College or above	529	9.2
Missing data	177	3.1
**Internet gaming disorder**		
No	5317	92.0
Yes	461	8.0

**Table 2.** Descriptive statistics and correlation^a^.

	Range	Mean (SD)	Student-student relationship, *r*	Student-staff relations, *r*	Academic emphasis, *r*	Shared values approach, *r*	School identification, *r*	Teacher-student relationship, *r*	Academic stress, *r*
**School climate**									
Student-student relationship	3‐15	11.7 (3.1)	1						
Student-staff relations	3‐15	11.8 (2.9)	0.55	1					
Academic emphasis	3‐15	11.8 (2.8)	0.51	0.83	1				
Shared values approach	3‐15	11.2 (3.2)	0.59	0.74	0.77	1			
School identification	3‐15	11.0 (3.3)	0.55	0.68	0.70	0.84	1		
Teacher-student relationship	0‐10	6.2 (1.9)	0.23	0.42	0.38	0.35	0.38	1	
Academic stress	0‐10	5.6 (2.9)	−0.19	−0.22	−0.19	−0.25	−0.26	−0.15	1
Anxiety	0‐21	4.0 (4.6)	−0.28	−0.31	−0.26	−0.33	−0.36	−0.21	0.41

a All correlation coefficients were of *P*<.001.

### Correlations

The 4 school climate subscales and the school identification subscale were positively correlated with teacher-student relationship (*r* ranged from 0.23 to 0.42) and negatively correlated with academic stress (*r* ranged from −0.26 to −0.19) and anxiety (*r* ranged from −0.36 to −0.26). Teacher-student relationship was negatively correlated with academic stress (*r*=−0.15) and anxiety (*r*=−0.21). Academic stress and anxiety were positively correlated with each other (*r*=0.41). All correlations among the 4 subscales of school climate and the school identification subscale were positive and of statistical significance (*r* ranged from 0.51 to 0.84) (see Table 2).

### Factors Associated With IGD

In the univariate analysis shown in Table 3, older students (crude odds ratio [ORc] 1.11, 95% CI 1.04-1.18), senior middle school students (vs junior middle school students, ORc 1.30, 95% CI 1.06-1.60), male students (vs female, ORc 2.63, 95% CI 2.07-3.34), and students not living with both parents (ORc 1.47, 95% CI 1.21-1.80) were more likely than others to have IGD. The associations involving hometown outside Taizhou, father’s educational level, and mother’s educational level were statistically nonsignificant.

**Table 3.** Background factors of internet gaming disorder.^a^

	ORc^b^ (95% CI)
Age	1.11 (1.04-1.18)^c^
**School type**	
Junior middle school	Reference=1.0
Senior middle school	1.30 (1.06-1.60)^d^
Vocational high school	1.40 (1.00-1.96)
**Gender**	
Female	Reference=1.0
Male	2.63 (2.07-3.34)^e^
**Living at the studied city constantly**	
Yes	Reference=1.0
No	1.09 (0.85-1.40)
**Living with both parents**	
Yes	Reference=1.0
No	1.47 (1.21-1.80)^e^
**Father’s educational level**	
Junior middle school or below	Reference=1.0
Senior middle school/vocational high school	0.85 (0.67-1.08)
College or above	0.70 (0.48-1.02)
**Mother’s educational level**	
Junior middle school or below	Reference=1.0
Senior middle school/vocational high school	0.97 (0.76-1.24)
College or above	0.70 (0.48-1.02)

a Missing data were excluded from the analysis.

b ORc: crude odds ratio.

c *P<.01.*

d *P<.05.*

e *P<.001.*

Similarly, the adjusted analysis presented in Table 4 showed that the 4 subscales of school climate (adjusted odds ratio [ORa] ranged from 0.87 to 0.88), school identification (ORa 0.85, 95% CI 0.83-0.88), and teacher-student relationship (ORa 0.80, 95% CI 0.76-0.84) were all negatively associated with IGD. Academic stress (ORa 1.18, 95% CI 1.14-1.23) and anxiety (ORa 1.16, 95% CI 1.14-1.18) were positively associated with IGD.

**Table 4.** Environmental, interpersonal, and individual factors of internet gaming disorder.^a^

	ORc^b^ (95% CI)	ORa^c^ (95% CI)
**School climate**		
Student-student relationship	0.88 (0.86-0.91)^d^	0.88 (0.85-0.91)^d^
Student-staff relations	0.87 (0.85-0.90)^d^	0.87 (0.84-0.90)^d^
Academic emphasis	0.89 (0.86-0.92)^d^	0.88 (0.85-0.91)^d^
Shared values approach	0.88 (0.85-0.90)^d^	0.88 (0.85-0.90)^d^
School identification	0.86 (0.83-0.88)^d^	0.85 (0.83-0.88)^d^
Teacher-student relationship	0.81 (0.77-0.85)^d^	0.80 (0.76-0.84)^d^
Academic stress	1.18 (1.14-1.23)^d^	1.18 (1.14-1.23)^d^
Anxiety	1.14 (1.12-1.16)^d^	1.16 (1.14-1.18)^d^

a The models were adjusted for background factors, including age, school types, gender, whether living in the studied city constantly, whether living with both parents, and father’s and mother’s educational level.

b ORc: crude odds ratio.

c ORa: adjusted odds ratio.

d *P*<.001.

### Testing Mediation Mechanisms by SEM

**Figure 1.**
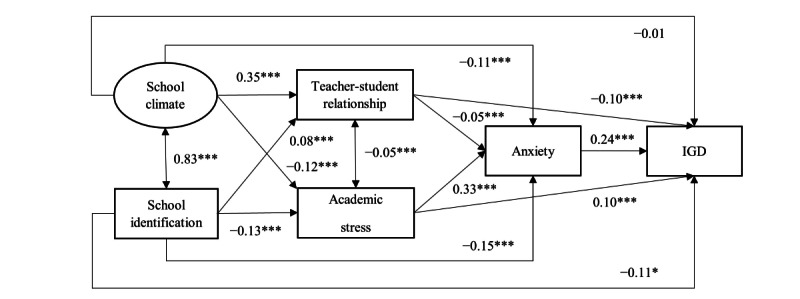
Structural equation modeling. Standardized coefficients were reported. The models were adjusted for background factors, including age, school types, gender, whether living in the studied city constantly, whether living with both parents, and father’s and mother’s educational level. IGD: internet gaming disorder. *P<.05, ***P<.001.

In the measurement model, the latent variable of school climate generated from the 4 subscales showed satisfactory goodness-of-fit indices (CFI=0.97; TLI=0.91; factor loadings ranged from 0.61 to 0.91, all *P*<.001), indicating that the latent variable was suitable for conducting SEM. Figure 1 presents the model testing the mediation effects of teacher-student relationship, academic stress, and anxiety between school climate or school identification and IGD, which also showed satisfactory goodness-of-fit indices (CFI=0.97; TLI=0.95; factor loadings of the latent variable ranged from 0.64 to 0.90, all *P*<.001).

In Figure 1, 3 single-mediator paths significantly mediated between school climate and IGD. (1) The significant mediation via perceived academic stress (*β=−*0.01; *P=.*004; mediation effect size=12.4%) indicated that school climate was negatively associated with perceived academic stress (*β=*−0.12; *P<.*001), which was in turn positively associated with IGD (*β=*0.10; *P*<.001). (2) The significant mediation via teacher-student relationship (*β=*−0.03*; P<*.001; mediation effect size=38.2%) indicated that school climate was positively associated with teacher-student relationship (*β=*0.35; *P*<.001), which was in turn negatively associated with IGD (*β=−*0.10; *P*<.001). (3) The significant mediation via anxiety (*β=*−0.03*; P*<.001; mediation effect size=29.2%) indicated that school climate was negatively associated with anxiety (*β=*−0.11; *P*<.001), which was in turn positively associated with IGD (*β*=0.24; *P*<.001). The 2 serial mediation paths between school climate and IGD were also statistically significant, indicating (1) mediations first via teacher-student and then via anxiety (β=−0.01; *P*<.001; mediation effect size=4.5%) and (2) mediations first via perceived academic stress and then via anxiety (β=−0.01; *P*<.001; mediation effect size=10.1%). All corresponding single-mediator paths and the serial mediation paths between school identification and IGD were also of statistical significance; the mediation effect sizes of the 5 paths were 7.2% (β=−0.01; *P*=.002), 4.2% (β=−0.01; *P*=.008), 20.4% (β=−0.03; *P*<.001), 0.6% (β=−0.01; *P*<.001), and 6.0% (β=−0.10; *P*<.001), respectively. In sum, a full mediation was found between school climate and IGD, as the direct path from school climate to IGD was statistically nonsignificant (β=−0.01; *P*=.91). Partial mediations were found between school identification and IGD, as the direct path was of statistical significance (β=−0.11; *P*=.03).

## Discussion

This study revealed the positive associations between both indicators of school environment (school climate at the environmental level and school identification at the individual level) and IGD among Chinese adolescents. Other individual-level (perceived academic stress and anxiety) and interpersonal (teacher-student relationship) factors were also determinants of IGD and significantly mediated the associations between school climate or school identification and IGD. These results support the multifaceted benefits of improving school climate and school identification and are implicative for future interventions reducing adolescent IGD.

This study found a prevalence of IGD of 8% (461/5778), which was slightly lower than that of 13% among junior middle school students in four Chinese cities in 2018 [12,49]. Geographical differences might exist. Since August 2021, China has enforced a policy confining adolescents’ access to online games only from 8 PM to 9 PM on Friday, Saturday, and Sunday [50]. It might have resulted in the lower IGD prevalence. The impact of this new policy has not been investigated; the speculation requires confirmation in future studies. IGD prevention in China is still needed, as the observed prevalence was higher than the 3.1% found among Australian adolescents aged 12 years or older [51] and the 3.5% among German adolescents aged 12‐17 years [52]. Such interventions should pay attention to the sociodemographic groups showing higher IGD prevalence in the present and previous studies, such as adolescents of the male sex [53,54] and those not living with both parents [54].

Corroborating previous studies [16-18], the adjusted logistic regression results showed that good school climate in terms of student-student relations, student-staff relations, academic emphasis, and shared values and approach were all significantly and negatively associated with IGD. Moreover, this study was the first one to reveal a negative association between school identification and IGD. The effect sizes (ORa) of the 5 subscales were comparable. Improvements in school climate and school identification are hence potentially useful for IGD prevention. Some researchers have given suggestions on how to improve school climate [55,56]. First, students should be given opportunities to participate actively in improving the learning and school environment. Second, parents need to understand, support, and engage in school activities, which would increase students’ emotional support for and engagement in schoolwork and build up stronger family-school connections addressing challenges related to academic performance and interpersonal relationships within the school environment. These would help enrich the school climate. Third, students should be provided with a wide range of opportunities and options to learn and put their learning into practice. Fourth, schools need to be a safe and caring social-emotional environment. Such orientations may also improve school identification. A higher level of participation in school activities was positively associated with school identification [57].

In general, the present study supports the socioecological model [25] inferring that IGD is determined by factors at environmental, interpersonal, and individual levels. Apart from school climate and school identification, significant individual-level factors (ie, perceived academic stress and anxiety) and an interpersonal factor (ie, teacher-student relationship) were identified. These findings corroborate the extant literature [18,27].

According to the Self Determination Theory, relatedness, along with autonomy and competence, is a determinant of health-related behaviors [58]. Students’ emotional connectedness with teachers is protective against risk behaviors in general [59] and IGD in particular [60]. To establish emotional connectedness, teachers need to create a helpful, caring, encouraging, and supportive learning environment [61]. Stress and anxiety could result in maladaptive behaviors, such as substance use and IGD [32,33]. Stress management skills should be introduced to students to reduce their academic stress and anxiety. Examples include guided imagery, mindfulness and meditation, support networking, and cognitive behavioral therapy. Notably, adolescents often use internet gaming as a means of stress reduction [62]; nonexcessive gaming could also be beneficial [63]. Yet, the intensity of gaming is a risk factor of IGD [64], and the gaming industry has instilled elements prolonging gaming that might induce addiction [65]. Thus, while academic stress and anxiety may lead to more intensive gaming for the purpose of stress reduction, the elevated intensity may result in IGD. In addition, although academic stress and anxiety may result in IGD, IGD may lead to poor academic performance and hence academic stress and anxiety [66]; bidirectional relationships between mental distress (eg, depression) and IGD have been reported [67]. A vicious cycle between anxiety, stress, and IGD might hence exist. Considering a poor teacher-student relationship is also a stressor, this “vicious cycle” perspective may also apply to understanding its relationship with IGD. However, this cross-sectional study could not discern causalities. Nonetheless, this study suggests that improvements in school climate or school identification would reduce all these 3 risk factors and IGD directly and indirectly and offers an insight that school-based interventions may break the potential vicious cycles.

The mediation analysis provides insights into how good school climate and school identification may protect adolescents from developing IGD via improvements in academic stress, teacher-student relationships, and anxiety. Given the relatively large sample size, some statistically significant mediations showed relatively small mediation effect sizes. Only 3 of the 10 paths had a mediation effect size exceeding 12%: (1) the 2 single-mediator paths involving teacher-student relationship (38.2%) and anxiety (29.5%) between school climate and IGD, and (2) the 1 involving anxiety (20.4%) between school identification and IGD. The mediation effect of academic stress might be smaller than those of teacher-student relationship and anxiety; the single-mediator effect and serial mediation effect involving academic stress were 12.4% and 10.1% between school climate and IGD, respectively, and only 7.2% and 6.0% between school identification and IGD, respectively. Notably, the mediation effects were stronger between school climate and IGD than those between school identification and IGD; the former involved full mediation while the latter involved partial mediation. The partial mediation implied that other unstudied mediators between school identification and IGD may exist and need to be explored.

Interestingly, school climate and school identification had independent effects on IGD, as both of them were significantly associated with IGD in the adjusted logistic regression analysis and the same model in SEM. A strength of this study is its use of a validated tool to assess school climate and school identification simultaneously. Both constructs need to be considered in IGD interventions, as their improvements may enhance the levels of the mediating protective factor (good teacher-student relationship) and reduce the levels of the mediating risk factors (perceived academic stress and anxiety), hence reducing the risk of IGD. The three mediators may not be easy to change and, as mentioned, some vicious cycles might occur. This study suggests a relatively new approach to modifying these factors by changing the school environment. While such interventions may attempt to reduce academic stress and anxiety directly, they may tackle the more fundamental issues of school climate and school identification. As the three mediators were associated with other mental health conditions (eg, depression) [26] and substance use [68], it is contended that the benefits of improvements in school climate and school identification would be multifaceted and not limited to IGD; the conjecture could be tested in future studies.

Apart from those mentioned, this study has several limitations. First, causal or temporal relationships could not be inferred due to the cross-sectional nature of the study design. Second, this study was conducted during the COVID-19 pandemic (February to March 2022), which might undermine the levels of school climate and school identification due to school suspension and online teaching. Notably, during the study period, zero new COVID-19 cases were found in Taizhou, and life in most Chinese cities, including Taizhou, was extremely normal without school closures and lockdowns. We hence believe that the impacts of the COVID-19 pandemic on school-related variables might be limited in this study. Third, as the study sample was conveniently selected from 9 schools in 1 Chinese city, the generalization of the results to other regions in China needs to be made cautiously. Efforts were made to reduce such selection bias by recruiting middle school students of various types and adjusting potential confounders in multivariable logistic regression and SEM (including age, school types, gender, whether living in the studied city constantly, whether living with both parents, and father’s and mother’s educational levels). Fourth, reporting bias may exist, including recall bias and social desirability bias. Fifth, the prevalence of IGD assessed by the 9-item *DSM-5* checklist might be overestimated when compared with that assessed by the *ICD-11* criteria of gaming disorder [69]. Sixth, the variables of both teacher-student relationship and academic stress were assessed by single-item scales. Nevertheless, such single-item scales have demonstrated good content validity and construct validity [70]. Last, the student-student relations subscale of the SCASIM-St15 represents the overall peer relationships within the school environment, but other peer variables associated with IGD (eg, peer support and peer influences) were not included in this study. Likely, the significance and direction of the association between student-student relations and IGD would change when taking into account other peer variables; future studies are warranted to confirm this speculation.

This study identified joint protective effects of school climate and school identification against IGD among Chinese adolescents. In addition, this study revealed the mediation effects of interpersonal factors of teacher-student relationship and individual-level factors of perceived academic stress and anxiety between school climate or school identification and IGD for the first time. Such findings underscore the important roles of the school environment in adolescent IGD. School-based interventions establishing good school climate and school identification might reduce the risk of IGD via improvements in protective and risk factors, including teacher-student relationships, academic stress, and anxiety. Interventions may consider these mechanisms. Future longitudinal and intervention studies are warranted to verify these findings and explore other potential mediators between school identification and adolescent IGD.

## Abbreviations

CFI: comparative fit index
*DSM-5*: *Diagnostic and Statistical Manual of Mental Disorders, Fifth Edition*
*ICD-11*: *International Classification of Diseases, 11th Revision*
IGD: internet gaming disorder
ORa: adjusted odds ratio
ORc: crude odds ratio
SCASIM-St15: 15-item Abbreviated Version of the Dual School Climate and School Identification Measure–Student
SCT: Social Cognitive Theory
SEM: structural equation modeling
TLI: Tucker-Lewis index
